# Cirevetmab, a novel canine anti–TGFβ monoclonal antibody, exerts antifibrotic effects on renal cells *in vitro*

**DOI:** 10.3389/fvets.2026.1885917

**Published:** 2026-09-04

**Authors:** Dayna L. Kappenman, Christopher L. Koehler, Reese N. Prentice, Nadia Koziar, Andrew Beardow, Alexandre Merlo, Christopher S. Knauer, Duncan Mwangi, Catrina Stirling

**Affiliations:** 1 Zoetis Inc., Global Therapeutics, Veterinary Medicine Research & Development, Kalamazoo, MI, United States; 2 PhenoVista Biosciences, San Diego, CA, United States; 3 Zoetis Inc., Global Portfolio Marketing, Parsippany, NJ, United States; 4 Zoetis Inc., Global Medical Affairs, Parsippany, NJ, United States; 5 Zoetis Inc., Global Regulatory Affairs, Veterinary Medicine Research & Development, Kalamazoo, MI, United States

**Keywords:** canine, chronic kidney disease, cirevetmab, fibrosis, monoclonal antibody, PSMAD, transforming growth factor β1

## Abstract

**Introduction:**

Transforming Growth Factor-β (TGF-β) has been implicated for its role in fibrosis and progression of chronic kidney disease (CKD) in both humans and rodents. Additionally, CKD is a common and progressive condition in canines, with striking pathological similarities to human disease. However, targeted therapies addressing fibrogenic mediators in dogs remain underexplored.

**Methods:**

We investigated the role of all three TGF-β isoforms (TGF-β1, TGF-β2, and TGF-β3) in CKD and renal fibrosis with *in vitro* model systems using human and canine primary renal proximal tubular epithelial cells. The studies assessed the induction of the canonical TGF-β pathway and the ability of a novel canine anti-TGF-β monoclonal antibody, cirevetmab, to neutralize these effects. All three TGF-β isoforms induced phosphorylation of SMAD3 (homologs of Drosophila mothers against decapentaplegic and C. elegans SMA proteins), nuclear translocation, and alpha-smooth muscle actin (αSMA).

**Results:**

Treatment with cirevetmab led to robust neutralization of TGF-β1-induced SMAD phosphorylation and fibrotic marker changes. Notably, although cirevetmab exerted modest neutralization of TGF-β3-induced SMAD phosphorylation, this was not associated with a significant change in TGF-β3-induced fibrotic markers. Conversely, no neutralization of TGF-β2-induced activity was observed with cirevetmab.

**Discussion:**

Our results demonstrate that cirevetmab selectively neutralizes TGF-β1 and, to a lesser extent, TGF-β3-induced SMAD phosphorylation *in vitro*, while only significantly neutralizing TGF-β1-induced fibrotic markers in renal proximal tubule cells.

## 1 Introduction

Chronic kidney disease (CKD) is a progressive disease characterized by the irreversible structural and/or functional impairment of one or both kidneys, persisting for more than 3 months. Unfortunately, structural damage often does not correlate with functional impairment, and overt clinical signs typically arise only after approximately 75% of functioning nephrons have been lost (1). CKD is the most common renal disease in elderly dogs, with reported prevalence estimates varying widely, reaching up to 15% of dogs over 10 years of age (2–5). CKD is currently considered incurable; available therapies slow progression or manage clinical signs rather than address the underlying pathophysiology of the disease.

Key pathological features of CKD include interstitial fibrosis, tubular atrophy, and tubulointerstitial inflammation. Persistent renal inflammation is pivotal to the development and progression of renal fibrosis (6–9). Renal fibrosis is the final common outcome for various types of renal disease and is characterized by excessive accumulation of extracellular matrix (ECM) proteins in the kidney, disrupting and replacing normal functional tissue (10–13). Initially noted in human health research, TGF-β is arguably the most important mediator in this process and often referred to as the master regulator in the pathogenesis of renal inflammation and fibrosis (7). It promotes the synthesis of ECM proteins while inhibiting their degradation, thereby advancing fibrotic pathology (7, 14). TGF-β drives fibrosis through both canonical and non-canonical pathways, ultimately facilitating the progression to end stage renal disease (ESRD).

In mammals, three TGF-β isoforms, TGF-β1, TGF-β2, and TGF-β3, are encoded by distinct genes and exhibit both tissue-specific and developmentally regulated expression patterns, with TGF-β1 being recognized as the predominant mediator in renal pathology (15). TGF-β is secreted as a latent complex consisting of an active TGF-β dimer, a dimer of latent associated peptide (LAP), and a latent TGF-β binding protein (LTBP) (16, 17). This latent complex is sequestered in the ECM until activation by factors such as integrins, thrombospondins, reactive oxygen species (ROS) and matrix metalloproteinases (MMPs) (18–20). Upon activation, TGF-β binds to its receptors, initiating downstream signaling via either canonical or non-canonical pathways, leading to nuclear translocation, and subsequently activating intracellular signaling and transcription of pro-fibrotic genes (19, 21–24).

**Figure 1:**
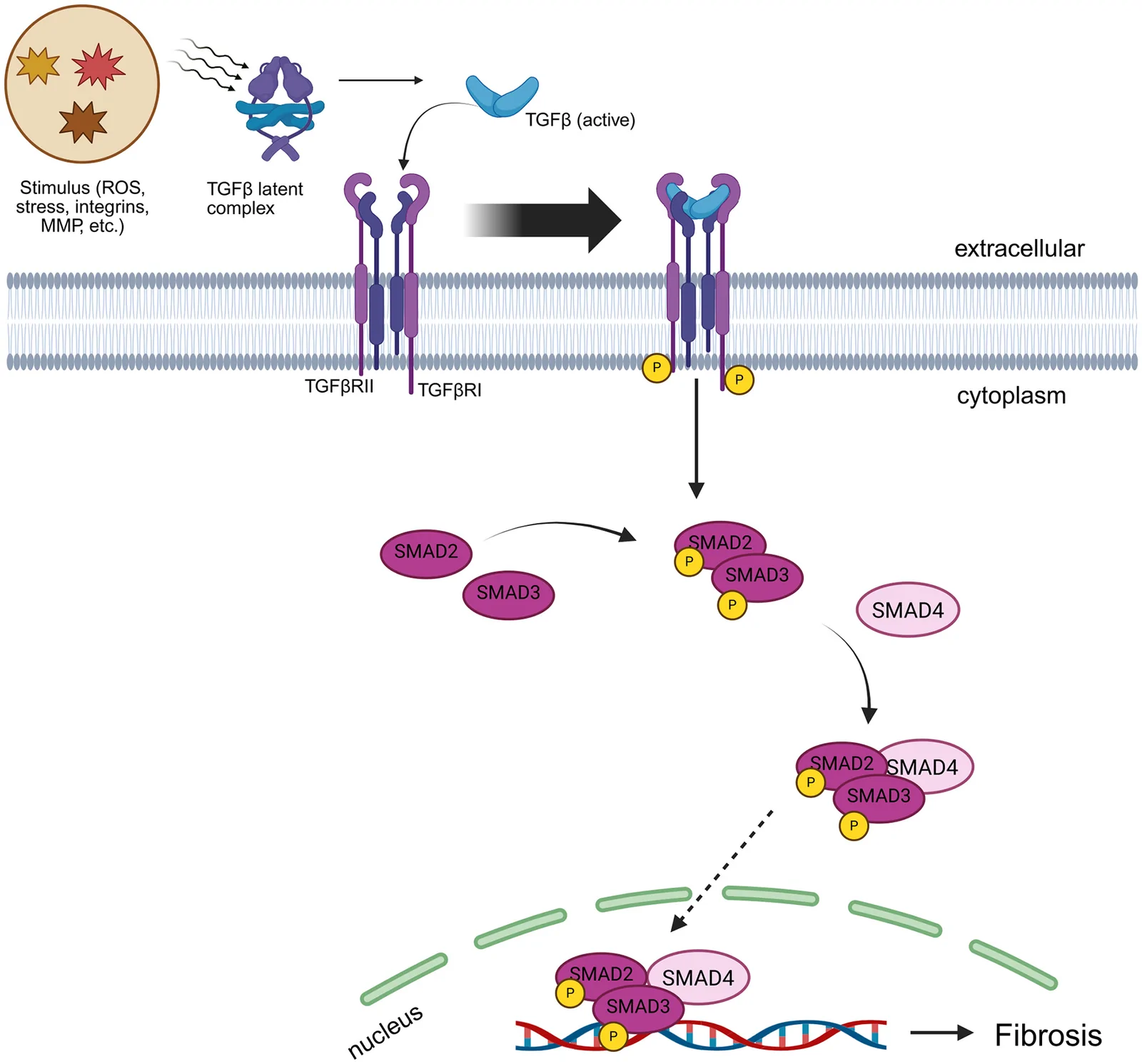
Schematic of TGF-β canonical signaling pathway. TGF-β is secreted as a latent complex and sequestered in the extracellular matrix until activation by any of several factors (ROS, integrins, MMPs, etc.). Upon activation, the active TGF-β binds to its receptors, initiating the signaling cascade. Formation of the receptor complex leads to phosphorylation of SMAD2/3 which oligomerizes with SMAD4. The SMAD complex then translocates to the nucleus, regulating transcription of profibrotic genes which can lead to fibrosis. Created in BioRender. Kappenman, (2026) https://BioRender.com/e6rme9v

The canonical pathway of TGF-β signaling is mediated by SMAD transcription factors. This pathway involves phosphorylation of intracellular TGF-βRI and TGF-βRII dimers to recruit, activate, and phosphorylate SMAD2 and SMAD3 proteins. The phosphorylated SMAD2/3 complex then associates with SMAD4 and is transported to the nucleus, where they regulate pro-fibrotic gene expression (18, 19, 25) (Figure 1). This intracellular signaling cascade directly contributes to fibrotic progression.

In addition to the canonical SMAD-dependent pathway, TGF-β can also signal through non-canonical, or SMAD-independent pathways. These involve activation of multiple signaling cascades, such as ERK, p38 MAPK, PI3/AKT, Rho guanosine triphosphate (GTPase), JNK, NFkB, and JAK/STAT which govern a diverse array of cellular processes including fibrosis, immunity, inflammation, metabolism, wound repair and tumorigenesis (19, 26). Fibrotic processes may be regulated independently through the non-canonical pathway or in cooperation with SMAD signaling (26). Both pathways play an important role in fibrosis; however, the mechanisms and conditions in which each pathway is activated are complex and highly context dependent (25, 27).

To investigate the canonical pathway of TGF-β-driven fibrosis in canine renal disease, we employed an *in vitro* model approach. We hypothesized that neutralization of TGF-β using a novel anti-TGF-β monoclonal antibody, cirevetmab, would inhibit SMAD2/3 phosphorylation, nuclear translocation, and ultimately suppress fibrotic processes. We evaluated TGF-β signaling and utilized *in vitro* models of renal fibrosis using proximal tubular epithelial cells to assess the canonical pathway across all three TGF-β isoforms. This multi-modal approach allowed us to establish mechanistic insights into TGF-β biology across species, while evaluating the antifibrotic potential of a canine-specific anti-TGF-β monoclonal antibody.

Despite the well-studied role of TGF-β in human renal fibrosis, its mechanisms in canine CKD remain less characterized. To our knowledge, this is the first *in vitro* investigation exploring TGF-β-mediated mechanisms in canine renal fibrosis and evaluation of a monoclonal antibody targeting TGF-β for antifibrotic effects in canine renal cells.

## 2 Materials and methods

To investigate the canonical TGF-β pathway and its role in canine renal fibrosis, we first established and optimized a cell-based assay using Crandell Rees Feline Kidney (CRFK) cells. This immortalized feline cell line served as a proof-of-concept model due to the high conservation of TGF-β isoforms across mammalian species. The robust signaling responses in CRFK cells allow for efficient assay development and optimization. We then translated these findings to primary canine renal proximal tubular epithelial cells (CaPTECs) as our model. Additionally, human renal proximal tubular epithelial cells (RPTECs) were used to establish species-specific correlations in TGF-β-driven pathology due to limited access to canine primary cells. Across all models, canonical TGF-β pathway activation was assessed by quantifying SMAD2/3 phosphorylation and profiling downstream fibrotic markers including α-SMA and actin fiber formation.

### 2.1 Reagents and materials

For pSMAD functional assay and epithelial-mesenchymal transition (EMT) visualization, recombinant human TGF-β1 and TGF-β3 were purchased from R&D Systems (Minneapolis, Minnesota, USA). Recombinant human TGF-β2 was purchased from PeproTech, Inc. (Cranbury, New Jersey, USA). Crandell-Rees Feline Kidney cell line (CRFK ATCC CCL-94) and Eagle's Minimum Essential Medium (EMEM) were purchased from American Type Culture Collection (ATCC) (Rockville, Maryland, USA). EMEM was supplemented with 10% equine serum (Hyclone™ Donor Equine Serum (US) from Cytiva (Marlborough, Massachusetts, USA). Canine Renal Proximal Tubular Epithelial Cells (CaPTECs), Epithelial Cell Medium kit and Primary Cells Detach kit were all purchased from Innoprot (Derio, Spain) for pSMAD functional assays. For epithelial-to mesenchymal transition (EMT) assays, adult human Renal Proximal Tubular Epithelial Cells (RPTECs), Human complete RenaEpi Growth Medium and Trypsin/EDTA subculture reagent kit were purchased from Cell Applications, Inc. (San Diego, California, USA). CaPTECs were purchased from Cell Biologics (Chicago, Illinois, USA). Other materials included Phosphate Buffered Saline (PBS) and 0.05% Trypsin-EDTA purchased from Life Technologies (Carlsbad, California, USA).

For nuclear translocation and phalloidin fiber formation assays, CaPTECs and complete epithelial medium were purchased from Cell Biologics (Chicago, Illinois, USA). Human primary renal proximal tubular epithelial cells and Renal Epithelial Growth Medium were purchased from Lonza Bioscience (Basel, Switzerland). Human recombinant TGF-β1, TGFβ-2, and TGFβ-3 were purchased from R&D Systems. Phospho-SMAD2 (Ser465/Ser467) Rabbit monoclonal antibody and Alexa Fluor^®^ 647 Phalloidin were purchased from Cell Signaling Technology (Danvers, Massachusetts, USA) and Anti-α-Smooth Muscle Actin (ACTA2) Antibody from Sigma-Aldrich (Burlington, Massachusetts, USA). Hoechst, Goat anti-Mouse Alexa Fluor^®^ 568 and Goat anti-Rabbit Alexa Fluor^®^ 568 Secondary Antibody were purchased from Fisher Scientific (Pittsburgh, Pennsylvania, USA).

For all assays, test and control antibodies, cirevetmab and ZTS-00116120 were produced and supplied by Zoetis (Zoetis Inc., Kalamazoo, Michigan, USA). Control antibody, 1D11, a TGF-β-1,2,3 antibody, was purchased from ThermoFisher Scientific and activin receptor-like kinase 5 inhibitor (ALK5i, a small molecule inhibitor of TGF-βRI kinase activity; Galunisertib) was purchased from Cayman Chemical Company (Ann Arbor, Michigan, USA) for nuclear translocation and phalloidin fiber formation assays.

### 2.2 pSMAD3 functional assay

Crandell-Rees Feline Kidney (CRFK) cells were maintained in complete growth medium (EMEM supplemented with 10% equine serum) at 37 °C in a humidified atmosphere of 5% CO_2_. When cell density reached ~85–90% confluency, cells were passaged using a 0.05% trypsin-EDTA solution. For assays, CRFK cells were seeded in 96-well tissue culture treated plates (Corning Inc., Corning, New York, USA) at 45,000 cells in a total volume of 100 μl per well in complete growth medium and allowed to grow for 12–16 h (overnight) at 37 °C/5% CO_2_. Cells were serum starved in EMEM for approximately 2 h prior to treatment. Recombinant human TGF-β1, TGF-β2, and TGF-β3 were titrated in serum-free EMEM with increasing concentrations from 0.034 pM to 2 nM to determine EC_80_ concentration. The Alpha Signal was plotted against TGF-β concentration using GraphPad Prism 10.2.2 for Windows (GraphPad Software, Boston, Massachusetts, USA) with a log(agonist) vs. response-variable slope (four parameters) least squares curve fit for EC_80_ determination. Cells were then treated with either 0.14 nM recombinant human TGF-β1, 0.5 nM recombinant human TGF-β2 or 0.06 nM recombinant human TGF-β3, based upon EC_80_ determinations, with increasing final concentrations of cirevetmab from 0.25 pM-15 nM for 30 min at 37 °C, then lysed in 1X AlphaLISA^®^ lysis buffer with a final volume of 25 μl per well. ZTS-00116120, a human-canine chimeric pan TGF-β antibody of GC1008 (fresolimumab) produced at Zoetis, was used as an assay control in all experiments. Fresolimumab has been demonstrated to bind all three active mammalian isoforms of TGF-β and block functional activity of TGF-β1, TGF-β2 and TGF-β3, therefore similar neutralization activity is expected with ZTS-00116120.

Primary CaPTECs from two separate healthy donors were maintained in complete Epithelial Cell Medium (Innoprot) in a poly-L-lysine coated flask (Greiner Bio-One, Kresmünster, Austria) at 37 °C in a humidified atmosphere of 5% CO_2_. When cells were >90% confluent they were lifted using trypsin/EDTA solution (Innoprot) and seeded in 96-well poly-l-lysine coated plates (Corning Inc.). Cells were seeded at 25,000 cells per well in a total volume of 100 μl in Epithelial Medium without FBS or antibiotics and returned to the incubator overnight. The next day, cells were treated with 0.06 nM recombinant human TGF-β1 with increasing concentrations of cirevetmab from 0.25 pM-15 nM final concentration in serum free/antibiotic free medium. Cells were treated for 45 min at 37 °C and lysed using 1X AlphaLISA^®^ lysis buffer in a final volume of 25 μl per well. For CaPTECs, only TGF-β1 was evaluated in the pSMAD3 assay using the CRFK-derived EC_80_ value. TGF-β2 and TGF-β3 were not tested in CaPTECs.

For both cell types, phosphorylated SMAD3 was quantified using the p-SMAD3 AlphaLISA^®^ *SureFire*^®^ *Ultra*™ detection kit purchased from Revitty, Inc (Waltham, Massachusetts, USA) per manufacturer's protocol. Results were obtained using Spectramax^®^ i3x (Molecular Devices, San Jose, California, USA) plate reader equipped with the AlphaScreen 384 Std Detection Cartridge.

The effects of test antibodies on TGF-β-induced pSMAD3 are expressed as percent neutralization relative to TGF-β only controls. The resulting percent neutralization data was plotted against antibody concentration using GraphPad Prism 10.2.2 for Windows (GraphPad Software, Boston, Massachusetts, USA) with a log(agonist) vs. response-variable slope (four parameters) least squares curve fit for IC_50_ determination.

### 2.3 TGF-β-induced nuclear translocation of pSMAD2 and fibrosis assays

CaPTECs (Cell Biologics) were maintained in Complete Epithelial Medium (Cell Biologics) at 37 °C with 5% CO_2_. Human RPTECs (Lonza Bioscience) were maintained in Renal Epithelial Growth Medium (Lonza Bioscience) at 37 °C with 5% CO_2_. One day prior to treatment, adherent cells were lifted from the TC-treated culture flask using TrypLE™ Express (Thermo Fisher Scientific). Cells were seeded into low-volume 384-well tissue culture treated imaging microplates (Greiner Bio-One, Kresmünster, Austria) that were pre-coated with poly-L-lysine (Sigma-Aldrich, Burlington, Massachusetts, USA). Seeding densities were 800 or 300 cells per well for the pSMAD2 translocation and αSMA/Phalloidin studies, respectively. Plates were incubated at 37 °C with 5% CO_2_ for 24 h prior to treatment.

TGF-β concentrations used in high-content imaging assays (pSMAD2 nuclear translocation and αSMA/phalloidin fiber formation) were selected empirically for each assay system and were not intended to represent EC_80_-equivalent conditions across cell types or across isoforms. Due to CaPTEC availability and proliferation constraints, TGF-β2- and TGF-β3-induced fibrotic marker changes in these high-content assays were evaluated in human RPTECs, whereas both CaPTECs and human RPTECs were assessed for TGF-β1-induced phenotypes.

Twenty-four hours after seeding, cells were pre-treated with cirevetmab or controls, ZTS-00116120, 1D11, and ALK5i. Treatments were diluted in the appropriate serum-free medium to 2X for the final treatment concentration, then administered via a one-half media exchange using an Apricot Designs Personal Pipettor (SPT Labtech, Melbourn, UK). For cirevetmab and ZTS-00116120, cells were treated with a 9-point dose response beginning with a high dose of 15 nM using a $\frac{1}{2}$-log dilution paradigm. 1D11 and ALK5i were administered at final, in well assay concentrations of 30 μg/ml and 10 μM, respectively. After addition of test articles, the cells were incubated for 30 min at 37 °C with 5% CO_2_ before stimulating with TGF-β.

For TGF-β stimulation, the CaPTECs were treated with recombinant human TGF-β1 protein (R&D Systems). The protein was administered via addition of 2.2 μL/well of a 10X stock solution prepared in serum-free medium. The final assay concentrations of TGF-β1 were 1 nM and 10 nM. The human RPTECs were treated with recombinant human TGF-β2 protein (R&D Systems) or recombinant human TGF-β3 (CHO expressed) protein (R&D Systems) via addition of 2.2 μL/well of a 10X stock solution prepared in serum-free medium. The final assay concentrations of TGF-β2 were 10 nM and 30 nM and for TGF-β3 were 3 nM and 30 nM. After addition of the TGF-β stimulation, cells were incubated for either 30 min before fixation to assess pSMAD2 nuclear translocation or 72 h prior to fixation to assess αSMA/Phalloidin fiber formation.

#### 2.3.1 Immunostaining

Following treatment of cells, assay medium was aspirated using an Apricot Designs Personal Pipettor and replaced with 4% paraformaldehyde (Santa Cruz Biotechnology, Dallas, Texas, USA) for 20 min at room temperature. Cells were permeabilized with Triton™ X-100 (Sigma-Aldrich) in PBS and blocked in PhenoVista Blocking Buffer (proprietary) overnight at 4 °C prior to immunostaining. Primary antibodies were diluted at 1:400 for Phospho-SMAD2 (Ser465/Ser467) Rabbit mAb (Cell Signaling Technology) or 1:200 for Anti-α-Smooth Muscle Actin (ACTA2) Antibody (Sigma-Aldrich) in PhenoVista blocking buffer. Primary antibodies were incubated overnight at 4 °C then washed with PBS to remove any unbound antibody. Secondary antibodies, phalloidin (a filamentous actin-specific dye), and Hoechst (a nuclear dye) were diluted in PBS and added to appropriate wells as follows. For both the pSMAD2 assay and αSMA/Phalloidin fiber formation assay, Hoechst (Fisher Scientific) was diluted 1:1,000, and Alexa Fluor^®^ 647 Phalloidin was diluted 1:400. For pSMAD2 assay, Goat anti-Rabbit Alexa Fluor^®^ 568 Secondary Antibody (Fisher Scientific) was used and diluted 1:1,000. For the αSMA/Phalloidin assay, Goat anti-Mouse Alexa Fluor^®^ 568 Secondary Antibody (Fisher Scientific) diluted 1:1,000. Cells were incubated with the secondary antibody/dye solutions for two h at room temperature before washing away unbound antibody/dye using serial washes of PBS. The final PBS wash was replaced with PBS supplemented with anti/anti (Cytiva, Marlborough, Massachusetts, USA) and Sodium Azide (Sigma-Aldrich) and sealed with AlumaSEAL (Excel Scientific, Victorville, California, USA).

#### 2.3.2 Image analysis

Assay plates were imaged using a Yokogawa CQ1 High Content Spinning Disc Confocal Scanner (Yokogawa, Tokyo, Japan) fitted with a 20X (UPLXAPO20X) objective using appropriate filter sets. Individual Z-slices from four fields per well were captured, and maximum intensity projections (MIPs) were generated. MIPs were analyzed for the relevant phenotypic metrics using custom-designed algorithms built in the Cell Pathfinder software suite (Yokogawa). Automated outlier detection was conducted using a custom Python script; individual data points greater than 1.5 IQR points above the third quartile or 1.5 IQR points below the first quartile were identified as outliers and removed from downstream analysis. Statistical significance versus control was determined via One-Way ANOVA followed by Dunnett's *post-hoc* test.

### 2.4 Epithelial to mesenchymal transition (EMT) assay

Adult human RPTECs (Cell Applications, Inc.) from a single healthy donor were cultured in Human Complete RenaEpi Growth Medium (Cell Applications) in a 37 °C, 5% CO_2_ humidified incubator. When cells were over 90% confluent, they were passaged using the recommended Trypsin/EDTA subculture reagent kit (Cell Applications) according to provided instructions. Cells were inoculated at 7,500 cells per cm^2^ and returned to the incubator until enough cells were obtained for assay.

For assay, human RPTECs were seeded in 96-well collagen Type I-treated plates (Corning Inc.) at 5,000 cells per well in a total volume of 100 μl in complete RenaEpi Growth Medium and returned to the incubator overnight. The following day, treatments were prepared in complete epithelial medium prior to addition to cells. TGF-β proteins were initially titrated with increasing concentrations from 0.034 pM-2 nM to determine concentrations that showed consistent morphological changes for each isoform. For TGF-β2, a “complete” morphological change was observed at ~1.7 nM. For both TGF-β1 and TGF-β3 the morphological changes were consistent with the previously determined EC_80_ concentrations in CRFK pSMAD3 functional assay, therefore concentrations were kept consistent for the EMT assay. To evaluate neutralization capabilities of cirevetmab on EMT, cells were treated with recombinant human TGF-β1 (0.6 nM), recombinant human TGF-β2 (2 nM) or recombinant human TGF-β3 (0.06 nM) in the presence of increasing concentrations of cirevetmab (0.25 pM-15 nM). Live-cell imaging was performed using an Incucyte^®^ S3 system (Sartorius, Göttingen, Germany), acquiring images every 3 h throughout the assay duration. Treatments were readministered every 2–3 days for 9 days.

Primary CaPTECs from a single healthy donor were purchased from Cell Biologics (Chicago, Illinois, USA) and propagated in complete epithelial cell medium (Cell Biologics) in a gelatin coated (Cell Biologics) culture flask at 37 °C, 5% CO_2_ humidified incubator. When cells were 100% confluent, they were passaged 1:2 using 0.05% Trypsin/EDTA solution (Cell Biologics) and returned to the incubator until enough cells were obtained for assay.

CaPTECs were seeded in gelatin-coated 96-well plates (Costar) at a density of 5,000 cells per well in a total volume of 100 μl complete epithelial cell medium and incubated overnight. The following day, treatments were prepared in complete epithelial cell medium and added to the cells. Due to limited cell availability and proliferation capabilities, TGF-β concentrations were kept consistent with those observed in the pSMAD3 functional assay. CaPTECs were treated with recombinant human TGF-β1 (0.14 nM), TGF-β2 (0.5 nM) or TGF-β3 (0.06 nM) with increasing concentrations of cirevetmab (0.25 pM-15 nM final concentration). Cell treatments were readministered every 2–3 days, and images were collected on Day 14 using an Incucyte^®^ S3 system.

## 3 Results

### 3.1 TGF-β-mediated SMAD3 phosphorylation functional assay

We utilized CRFK cells to establish the ability of all three TGF-β isoforms to induce SMAD phosphorylation, using AlphaLISA^®^ technology for quantitation. Of note, the active forms of human and canine TGF-β share 100% sequence identity; therefore, human recombinant proteins were used in all experiments, due to their availability and cost-effectiveness.

**Figure 2:**
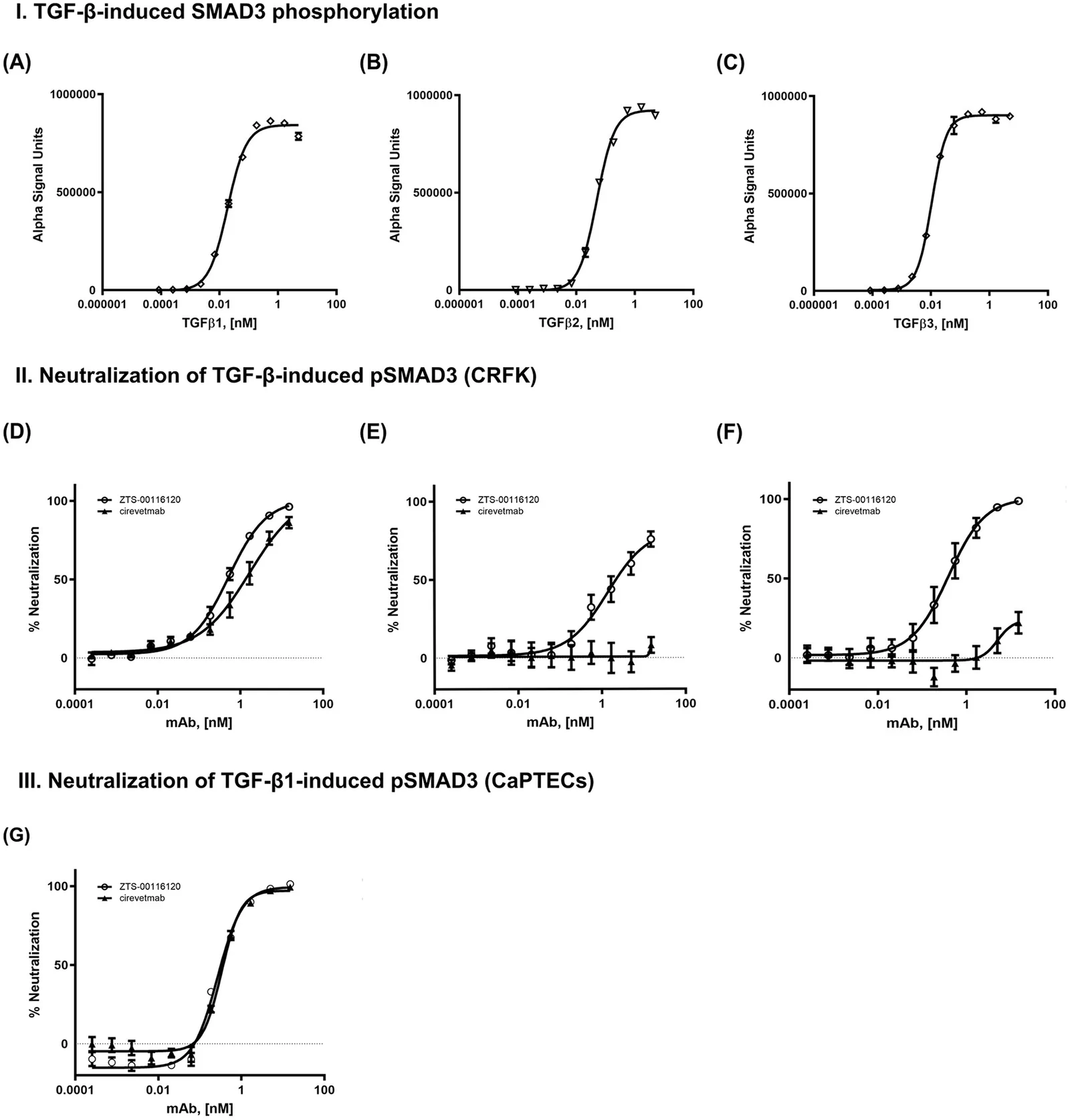
Series of experiments to demonstrate the effects of human TGF-β1, TGF-β2, and TGF-β3 on SMAD3 phosphorylation and the ability of cirevetmab to selectively neutralize in both CRFK cells and primary canine proximal tubule epithelial cells. I. TGF-β-induced SMAD3 phosphorylation.TGF-β1 **(A)**, TGF-β2 **(B)**, and TGF-β3 **(C)** induce dose dependent phosphorylation of SMAD3 in Crandell-Rees Feline Kidney (CRFK) cells. EC80 concentrations for TGF-β1, TGF-β2, and TGF-β3 were determined to be 0.05 nM, 0.38 nM, and 0.02 nM respectively. Representative curves shown, error bars = Standard Error of the Mean, *n* = 2. II. Neutralization of TGF-β-induced SMAD3 (CRFK). Canine anti-TGF-β monoclonal antibody, cirevetmab, neutralizes **(D)** TGF-β1-induced phosphorylation of SMAD3 in Crandell-Rees Feline Kidney (CRFK) cells in a dose dependent manner with an IC50 concentration of 1.7 nM. Cirevetmab had no effect on **(E)** TGF-β2-induced SMAD3 phosphorylation and showed reduced potency against **(F)** TGF-β3-induced pSMAD at the concentrations tested. ZTS-00116120, a human-canine chimeric pan anti-TGF-β monoclonal antibody, showed dose dependent neutralization of all three TGF-β isoforms. Representative curves shown, error bars = Standard Error of the Mean, *n* = 6. III. Neutralization of TGF-β1-induced pSMAD3 (CaPTECs). **(G)** Canine anti-TGF-β monoclonal antibody, cirevetmab, neutralizes TGF-β1-induced phosphorylation of SMAD3 in canine primary proximal tubule epithelial cells (CaPTECs) in a dose dependent manner with an IC50 concentration of 0.7nM. ZTS-00116120, a human-canine chimeric pan anti-TGF-β monoclonal antibody demonstrated similar neutralization capabilities. Average curves shown, two donors, error bars= Standard Error of the Mean, *n* = 12.

The concentration of each TGF-β isoform required to elicit a maximal cellular response, and concentrations producing 80% of maximal activity (EC_80_) were first determined to guide subsequent experiments. CRFK cells demonstrated robust, dose-dependent phosphorylation of SMAD3 in response to all three TGF-β isoforms. EC_80_ values, calculated from the mean of six independent dose-response titration curves, were determined to be 0.05 nM, 0.38 nM, and 0.02 nM for TGF-β1, TGF-β2 and TGF-β3 respectively (Figure 2).

The neutralizing capabilities of cirevetmab were evaluated by preincubating the antibody with each TGF-β prior to adding to the cells. ZTS-00116120 served as a positive control throughout all experiments. Cirevetmab completely neutralized TGF-β1-induced SMAD3 phosphorylation in CRFK cells, with an average IC_50_ value of 1.7 nM ± 0.63, comparable to that of the control antibody. In contrast, cirevetmab did not neutralize TGF-β2-induced SMAD3 signaling. For TGF-β3, only partial neutralization was observed with cirevetmab at the tested concentration range, and its potency was markedly reduced relative to both TGF-β1 and the control antibody (Figure 2).

To assess potential species-specific differences, the same procedure was performed using CaPTECs. Due to the limited cell availability and proliferation potential, these experiments were restricted to TGF-β1 stimulation using cells from two independent healthy canine donors and the EC_80_ concentration determined in the CRFK assay was utilized. Both cirevetmab and the control antibody demonstrated comparable neutralizing activity against TGF-β1-induced SMAD3 phosphorylation with an average IC_50_ value of approximately 0.7 nM for each, in agreement with the results observed in CRFK cells (Figure 2).

### 3.2 TGF-β-induced nuclear translocation of pSMAD2 and fibrosis assays

Human and canine primary proximal renal tubular epithelial cells were utilized to assess the functional potency of cirevetmab via high content imaging assays. These assays quantified phenotypic responses to TGF-β stimulation, including pSMAD2 nuclear translocation and αSMA/Phalloidin fiber formation, ensuring robust and measurable results (see Materials and Methods). Fibrotic phenotypes were characterized by increases in pSMAD2 nuclear translocation, which is indicative of TGF-β pathway activation, and by increases in the formation of F-actin and α-SMA-containing fibers, hallmarks of tissue stiffening in fibrosis.

**Figure 3:**
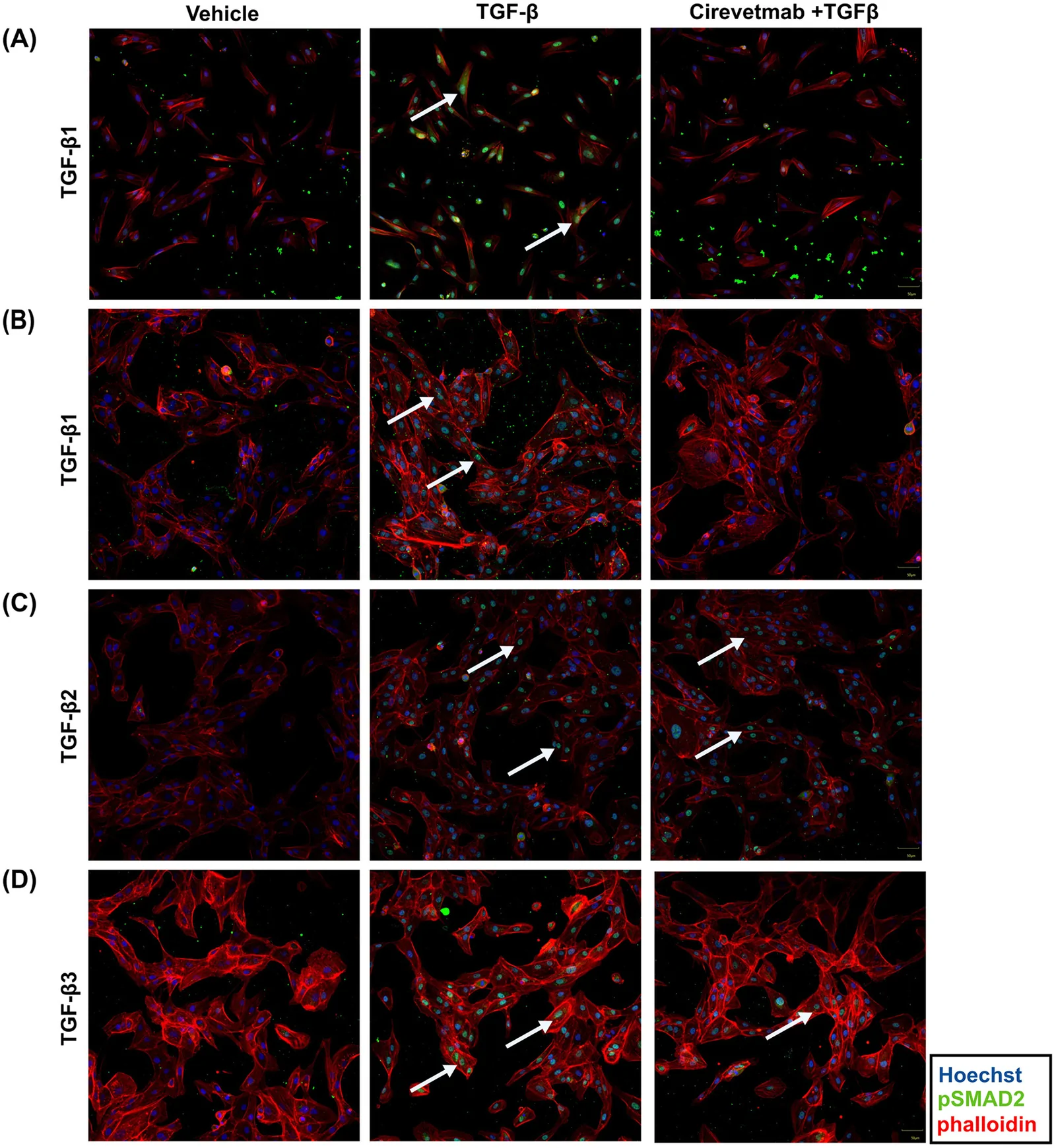
TGF-β-induced nuclear translocation of pSMAD2. TGF-β pathway activation was assessed via visualizing pSMAD2 nuclear translocation using high content imaging. **(A)** Canine proximal tubule epithelial cells (CaPTECs) stimulated with 1 nM TGF-β1 showed increased nuclear translocation of pSMAD2 as indicated by arrows. Treatment with 15 nM cirevetmab significantly neutralized nuclear pSMAD2. **(B)** Human renal proximal tubule cells (RPTECs) treated with 10 nM TGF-β1 resulted in increased pSMAD2 nuclear translocation as indicated by arrows. Treatment with cirevetmab significantly inhibited nuclear translocation. **(C)** Human proximal tubule cells treated with 10 nM TGF-β2 resulted in increased pSMAD2 nuclear translocation as indicated by arrows. Treatment with cirevetmab failed to show inhibitory effects. **(D)** Human proximal tubule cells treated with 10 nM TGF-β3 promoted increased pSMAD2 nuclear translocation as indicated by arrows. Cirevetmab showed no significant effects across concentrations tested.

We first assessed pathway activation via pSMAD2 nuclear translocation. Upon stimulating CaPTECs with 1 nM TGF-β1, cells consistently exhibited pathway activation. Both the positive assay controls and cirevetmab significantly inhibited TGF-β1-induced pSMAD2 nuclear translocation, measured 30 min after stimulation. Cirevetmab and ZTS-00116120 showed significant neutralizing ability across the entire tested concentration range. These effects were reflected in measurements of the total nuclear pSMAD2 intensity per cell with IC_50_ values of 1.99 nM for cirevetmab and 3.95 nM for ZTS-00116120 (Figure 3).

Due to limited availability and proliferation capabilities of CaPTECs, the ability of cirevetmab and ZTS-00116120 to inhibit fibrotic phenotypes induced by TGF-β1 (10 nM) in human RPTECs was assessed to gain a better understanding of species translatability. These antibodies were tested in parallel with controls Alk5i (10 μM) and 1D11 (30 μg/ml). Fibrotic phenotypes were characterized as described for canine cells.

As observed in CaPTECs, stimulation of human RPTECs with TGF-β1 resulted in increased pSMAD2 nuclear translocation. Treatment with cirevetmab showed inhibition of pSMAD nuclear translocation with an IC_50_ value of 16.8 nM (Figure 3). These results were comparable to those observed with the CaPTECs, albeit higher likely due to experimental differences (i.e. higher TGF-β1 concentration), and gave confidence in using human RPTECs moving forward to evaluate the TGF-β2 and TGF-β3 effects.

TGF-β2-induced pSMAD2 nuclear translocation was assessed in human RPTECs at both 10 nM and 30 nM concentrations. Stimulation with TGF-β2 resulted in similar changes with both tested concentrations; however, the fibrotic signatures were only effectively inhibited by Alk5i and 1D11 with the 10 nM dose of TGF-β2. At 30 nM TGF-β2, 1D11 showed no preventative effect, but Alk5i remained effective. Pre-treatment of human RPTECs with 15 nM ZTS-00116120 prevented pSMAD2 nuclear translocation induced by TGF-β2, represented by nuclear pSMAD2 intensity per cell. Cirevetmab, in contrast, failed to show any clear inhibitory effects at the tested concentrations (Figure 3).

The ability of cirevetmab and ZTS-00116120 to inhibit TGF-β3-induced fibrotic phenotype in human RPTECs was likewise evaluated, in parallel with controls Alk5i and 1D11. As with earlier results, stimulation of human RPTECs with TGF-β3 promoted pSMAD2 nuclear translocation which was significantly prevented by treatment with Alk5i and 1D11. ZTS-00116120 also significantly inhibited TGF-β3-induced nuclear translocation in a dose-dependent manner, while cirevetmab showed no significant effects at concentrations tested (Figure 3).

**Figure 4:**
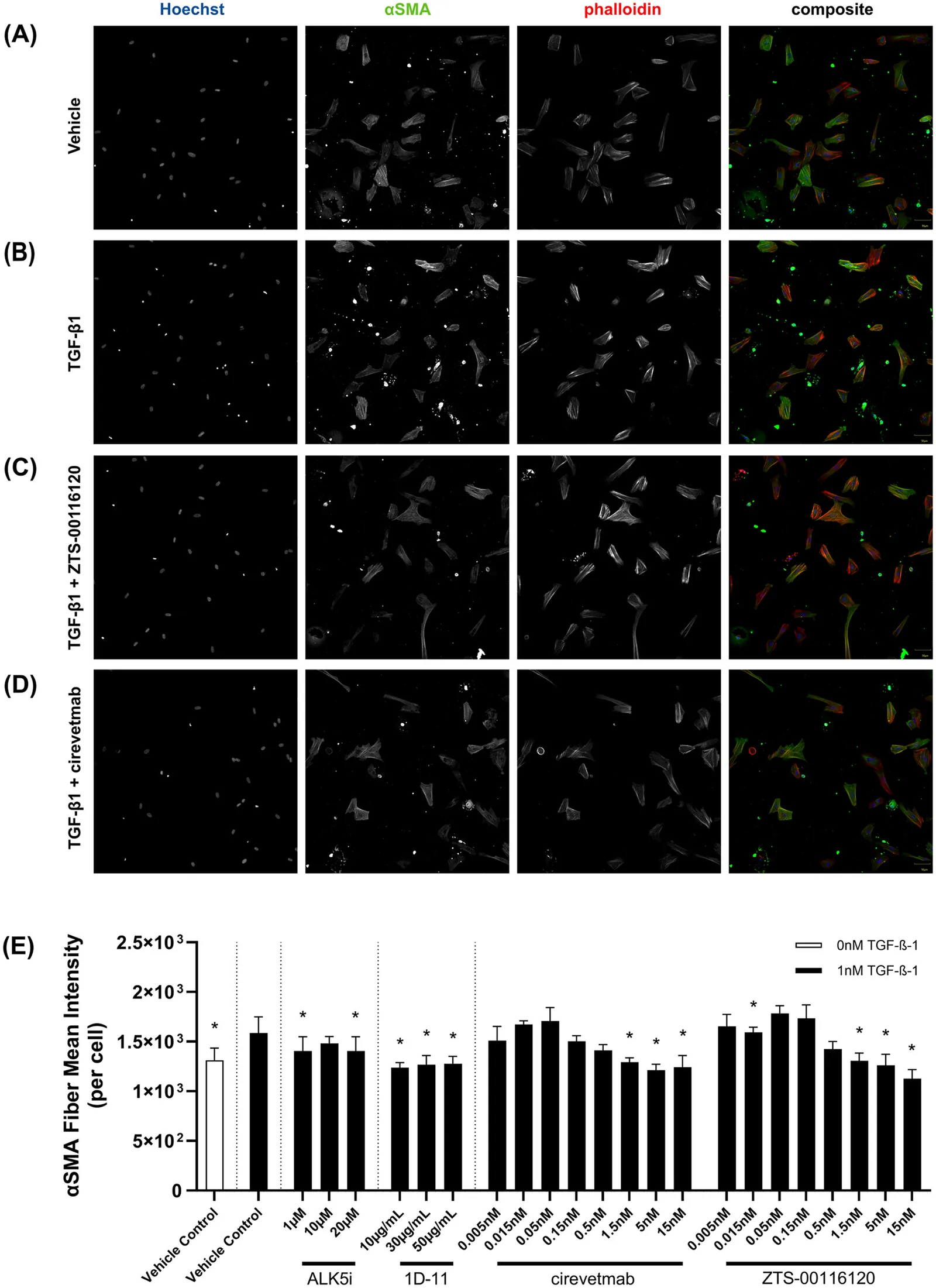
TGF-β1 induces fibrotic phenotypes in canine proximal tubule epithelial cells. Fibrotic phenotypes were evaluated via F-actin and α-SMA fiber formation in CaPTECs using high content imaging. **(A)** Untreated cells show low levels of α-SMA and phalloidin, which is upregulated when treated with 1 nM TGF-β1 **(B)**. **(C, D)** Demonstrate inhibition of both α-SMA and phalloidin when treated with ZTS-00116120 and cirevetmab respectively. The IC50 for Cirevetmab was 0.47 nM and for ZTS-00116120 was 0.88 nM, showing comparable potency in this assay. **(E)** Significant inhibition of fibrotic phenotypes was observed with cirevetmab and controls. *indicates statistical significance vs TGFb-stimulated Vehicle Control.

To further evaluate fibrotic phenotypes, F-actin and α-SMA fiber formation were measured. TGF-β1-induced changes in F-actin and α-SMA fiber formation in CaPTECs were significantly inhibited by cirevetmab and ZTS-00116120 along with assay controls Alk5i and 1D11. Cirevetmab and ZTS-00116120 showed comparable potency in terms of IC_50_ with values of 0.47 nM and 0.88 nM respectively (Figure 4).

**Figure 5:**
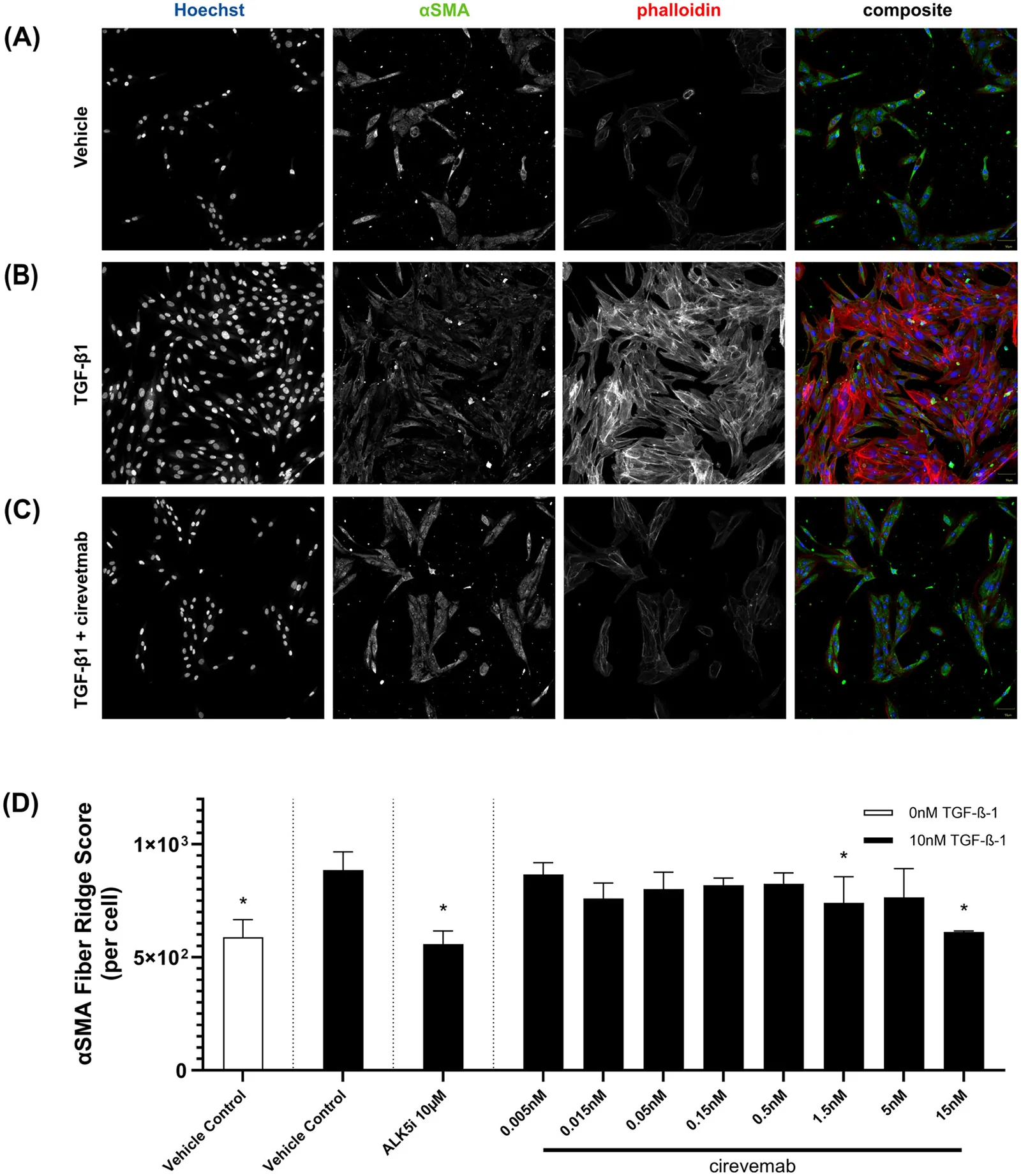
TGF-β1 induces fibrotic phenotypes in human proximal tubule epithelial cells. Fibrotic phenotypes were evaluated via F-actin and α-SMA fiber formation in human RPTECs using high content imaging. **(A)** Untreated cells show low levels of α-SMA and phalloidin, which is upregulated when treated with TGF-β1 **(B)**. **(C)** Demonstrates inhibition of both α-SMA and phalloidin when treated with cirevetmab. **(D)** The IC50 for Cirevetmab was 47.7 nM and showed significant inhibition of TGF-β1-induced phenotypes. ^*^indicates statistical significance vs TGFb-stimulated Vehicle Control.

Again, human RPTECs were used to evaluate TGF-β1-induced F-actin and α-SMA fiber formation due to the cell constraints for canine previously mentioned. These metrics were significantly inhibited by cirevetmab with an IC_50_ of 47.7 nM (Figure 5). These results were aligned with expected results based upon differences in TGF-β1 concentrations, therefore human RPTECs were used moving forward to evaluate TGF-β2 and TGF-β3.

**Figure 6:**
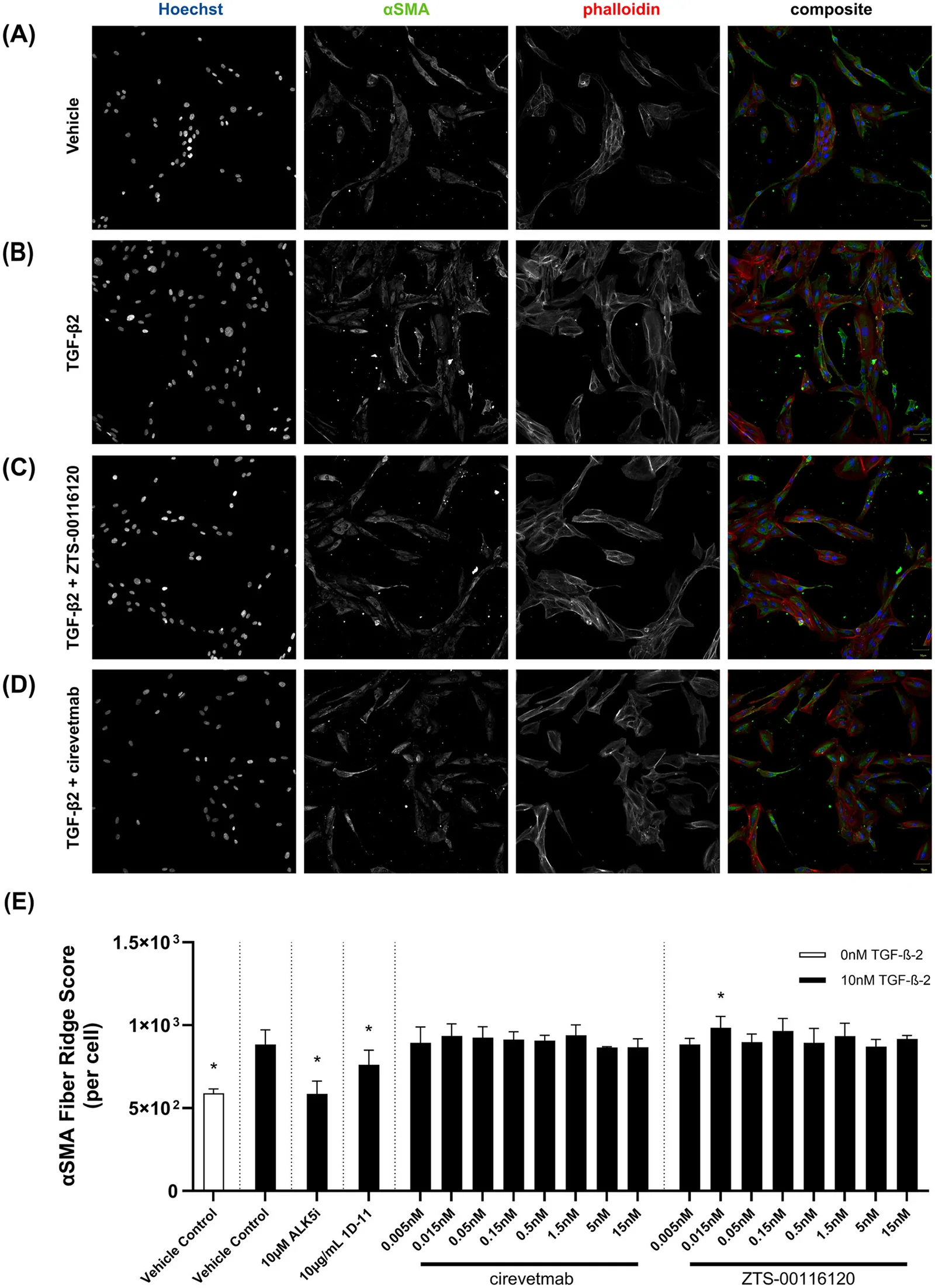
TGF-β2 induces fibrotic phenotypes in human proximal tubule epithelial cells. Fibrotic phenotypes were evaluated via F-actin and α-SMA fiber formation in human RPTECs using high content imaging. **(A)** Untreated cells show low levels of α-SMA and phalloidin, which is upregulated when treated with 10 nM TGF-β2 **(B)**. **(C)** Demonstrates inhibition of both α-SMA and phalloidin when treated with ZTS-00116120 but not cirevetmab **(D)**. **(E)** Significant inhibition of fibrotic phenotypes was observed with controls but not cirevetmab. *indicates statistical significance vs TGFb-stimulated Vehicle Control.

TGF-β2-induced F-actin and α-SMA fiber formation changes were induced with both 10 nM and 30 nM concentrations; however, inhibition was only observed with the 10 nM dose of TGF-β2 by the controls. Alk5i also was effective with the 30 nM TGF-β2 treatment. Pre-treatment of human RPTECs with 15 nM ZTS-00116120 prevented F-actin and α-SMA fiber formation. Cirevetmab, in contrast, failed to show any clear inhibitory effects against TGF-β2-induced changes at the tested concentrations (Figure 6).

**Figure 7:**
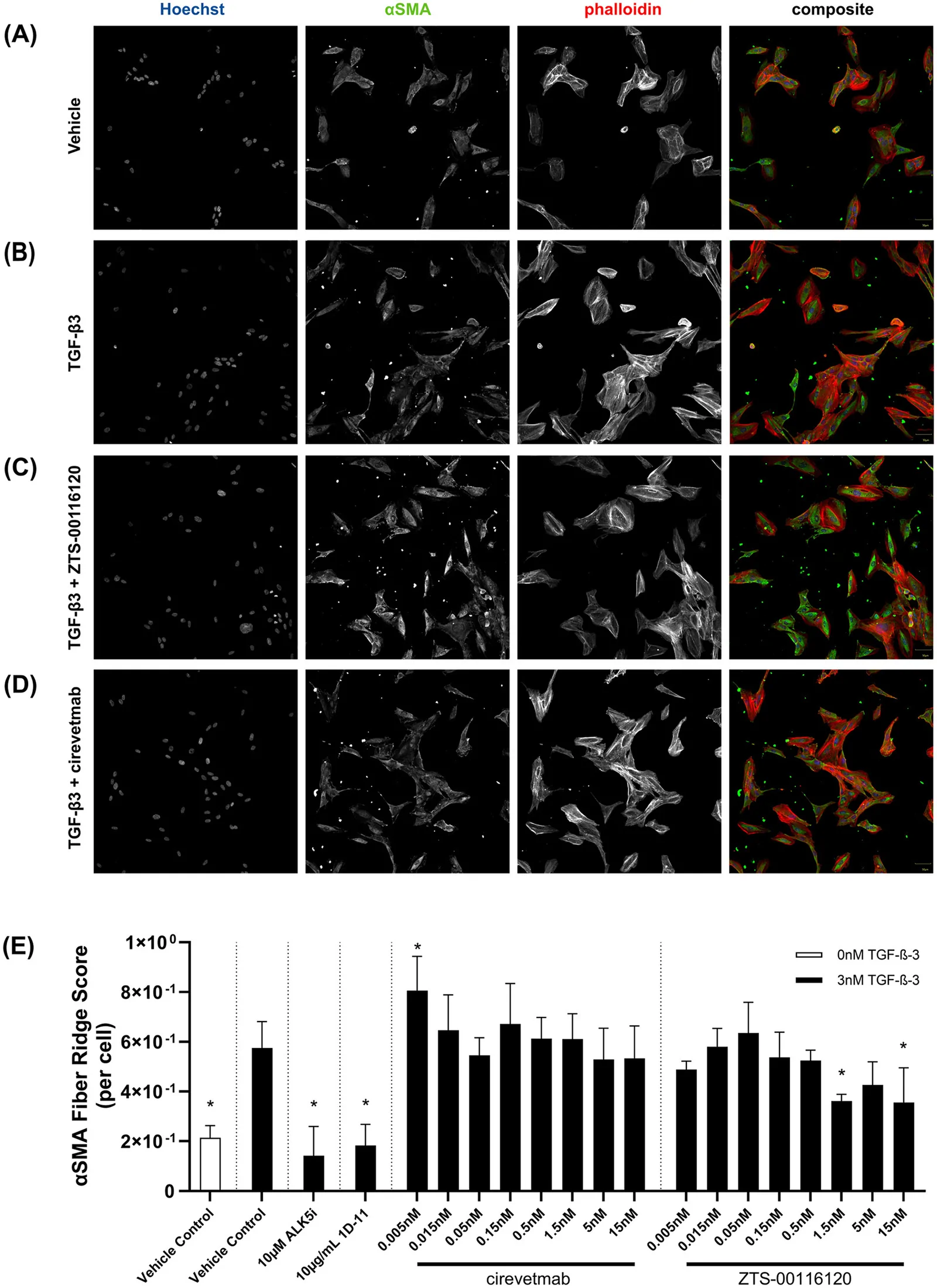
TGF-β3 induces fibrotic phenotypes in human proximal tubule epithelial cells. Fibrotic phenotypes were evaluated via F-actin and α-SMA fiber formation in human RPTECs using high content imaging. **(A)** Untreated cells show low levels of α-SMA and phalloidin, which is upregulated when treated with 3 nM TGF-β3 **(B)**. **(C)** Demonstrates inhibition of both α-SMA and phalloidin when treated with ZTS-00116120 but not cirevetmab **(D)**. **(E)** Significant inhibition of fibrotic phenotypes was observed with ZTS-00116120 at the highest doses and controls Alk5i and 1D11, but not cirevetmab. *indicates statistical significance vs TGFb-stimulated Vehicle Control.

The ability of cirevetmab and ZTS-00116120 to inhibit TGF-β3-induced F-actin and α-SMA fiber formation was likewise evaluated in human RPTECs at concentrations of 3 nM and 30 nM. As in previous results, stimulation of human RPTECs with TGF-β3 promoted increased levels of F-actin and α-SMA fibers. With 3 nM TGF-β3 treatment, high doses of ZTS-00116120 significantly prevented increases in F-actin and α-SMA fiber formation, while cirevetmab had no effect. With 30 nM TGF-β3 treatment, the antibodies tested did not have inhibitory effects (Figure 7).

In summary, cirevetmab effectively neutralized TGF-β1-induced fibrotic phenotypes, restoring levels to near baseline activity, in line with the control antibody. Notably, cirevetmab did not show neutralization of TGF-β2-induced effects, consistent with CRFK cell functional data. Furthermore, in agreement with pSMAD signaling observed via AlphaLISA^®^, in human RPTECs, cirevetmab showed no significant neutralization of TGF-β3-induced pSMAD2 nuclear translocation or F-actin and α-SMA fiber formation at the concentrations tested. Because isoform-specific dose-response relationships were not established within each model used here (TGF-β2 and TGF-β3 fibrosis assays were performed using human RPTECs due to CaPTEC constraints), these findings should be interpreted as describing cirevetmab activity under the specific ligand concentrations and assay conditions tested.

### 3.3 Visualization of TGF-β-mediated epithelial to mesenchymal transition assay

**Figure 8:**
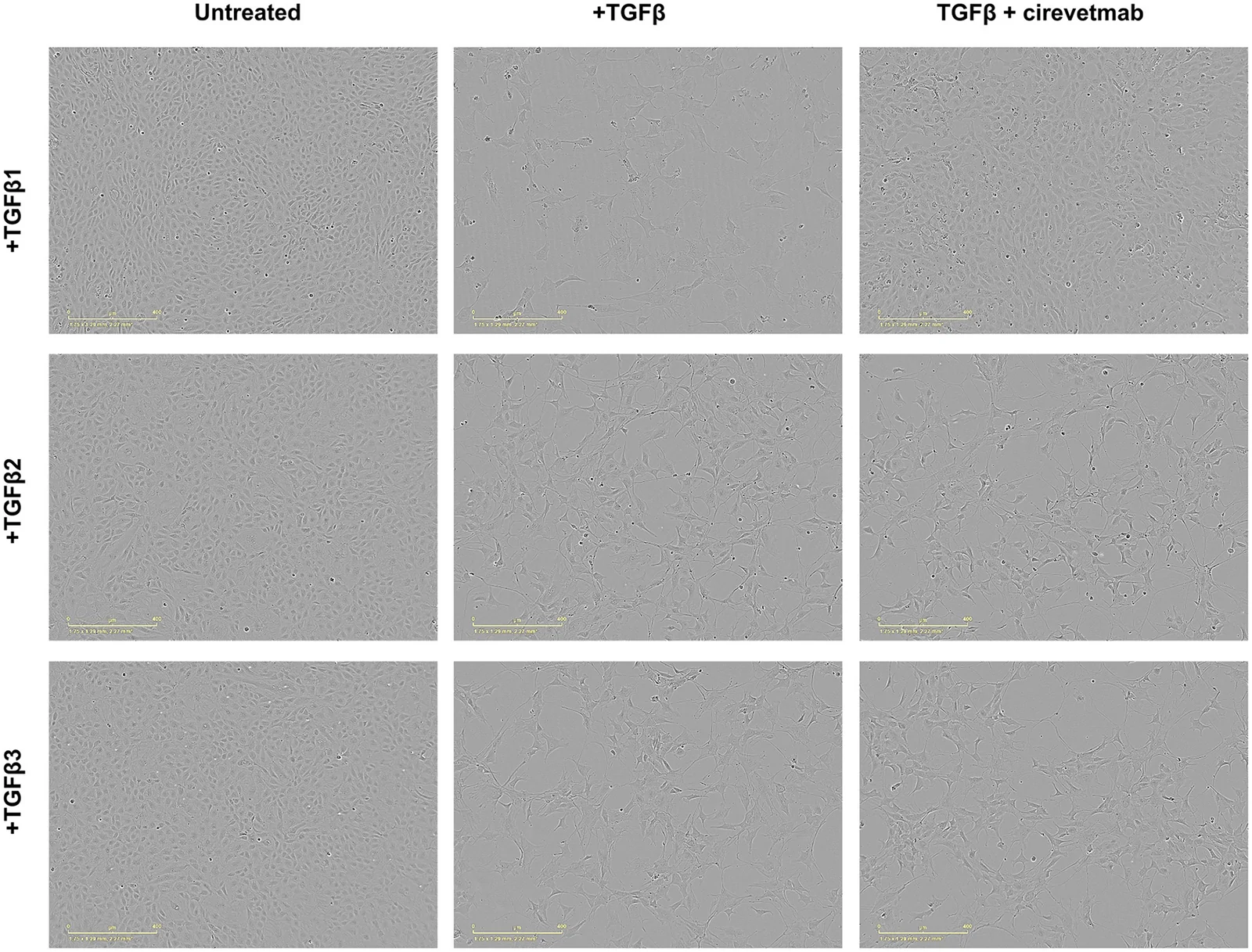
Morphological changes in human primary proximal tubule epithelial cells were observed following TGF-β treatment. Human primary proximal tubule epithelial cells (RPTECs) were treated with recombinant TGF-β1 (0.06 nM), TGF-β2 (2 nM) or TGF-β3 (0.06 nM) as shown. Regardless of isoform, TGF-β treatment resulted in visual morphological changes resulting in a transition from tightly packed, cobblestone epithelial morphology (untreated) to elongated, spindle-like, and scattered appearance consistent with mesenchymal cells. Treatment with 15 nM cirevetmab appeared to largely attenuate observed morphological changes associated with TGF-β1 treatment, but not TGF-β2 and only partial mitigation of TGF-β3 following 9 days of treatment.

We used human primary proximal tubule epithelial cells to visualize morphological changes associated with TGF-β treatment. Treatment with TGF-β, regardless of the isoform, resulted in a marked transition from the characteristic tightly packed, cobblestone epithelial morphology, to an elongated, spindle-like, and scattered appearance consistent with that of mesenchymal cells. These morphological changes were observed in a dose-dependent manner for all three isoforms of TGF-β (qualitative; data not shown). Consistent with our previous data, the addition of cirevetmab to the culture showed markedly reduced morphological changes associated with TGF-β1 treatment, whereas no preservation of epithelial morphology was observed with TGF-β2, and the effects of TGF-β3 appeared only partially mitigated (Figure 8).

**Figure 9:**
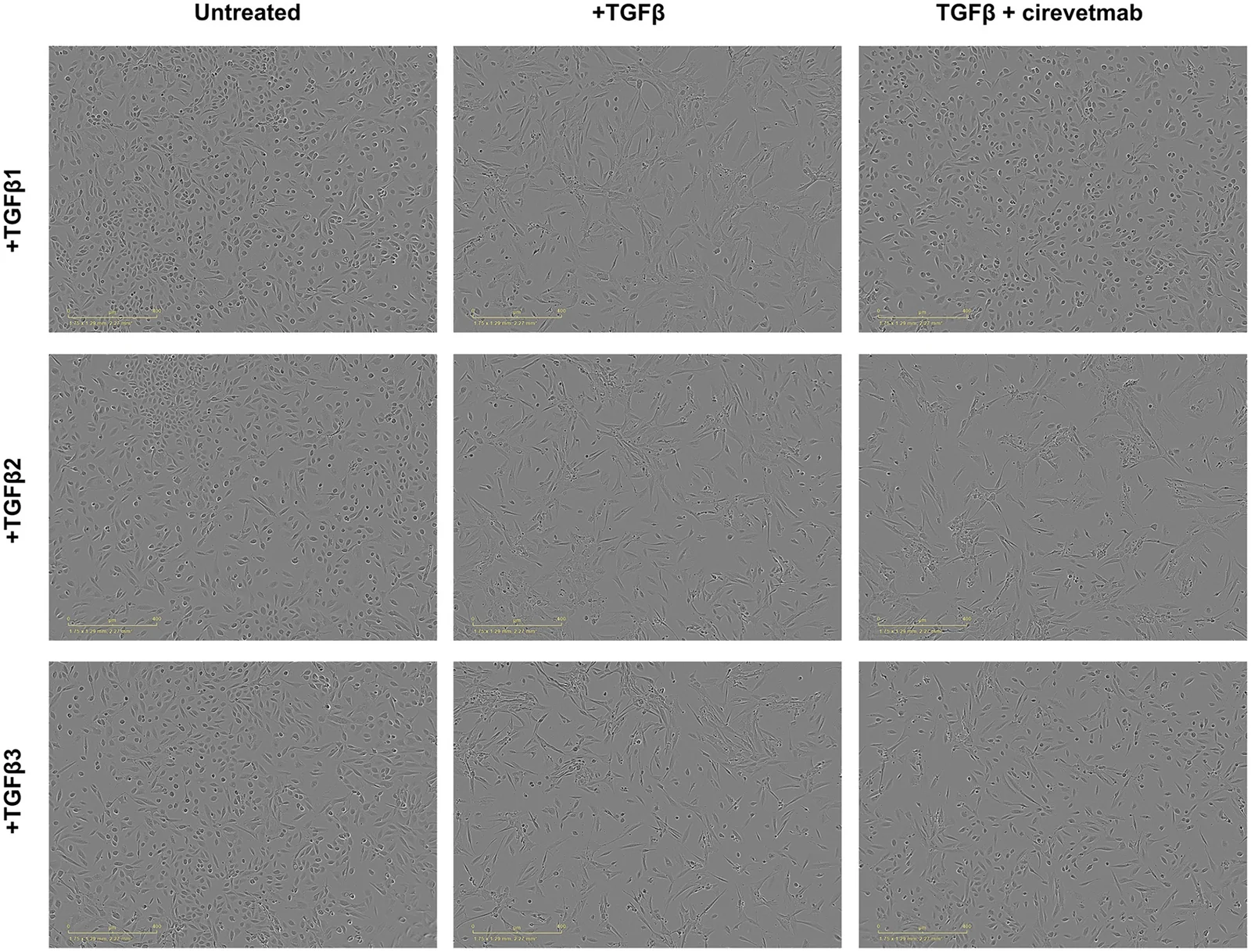
Morphological changes in canine primary proximal tubule epithelial cells were observed following TGF-β treatment. Canine primary proximal tubule epithelial cells (CaPTECs) were treated with recombinant TGF-β1 (0.14 nM), TGF-β2 (0.5 nM), or TGF-β3 (0.06 nM) as shown. Regardless of isoform, TGF-β treatment resulted in observed morphological changes resulting in a transition from tightly packed, cobblestone epithelial morphology (untreated) to elongated, spindle-like, and scattered appearance consistent with mesenchymal cells. Treatment with 15 nM cirevetmab showed qualitative reduction of morphological changes associated with TGF-β1 treatment, but not TGF-β2 or TGF-β3 following 14 days of treatment.

Similar experiments with canine primary proximal tubule epithelial cells revealed comparable TGF-β-induced phenotypic changes, underscoring a correlation between human and canine cellular responses. Treatment with TGF-β1, TGF-β2, or TGF-β3 resulted in distinct mesenchymal-like morphological changes. In agreement with other *in vitro* data, addition of cirevetmab to culture appeared to neutralize TGF-β1-induced changes. Morphology was not preserved, even at the highest concentrations of cirevetmab tested, for cells treated with TGF-β2 or TGF-β3, consistent with high content imaging data for markers of fibrosis (Figure 9). A limitation of this experiment is that the morphological transition was evaluated qualitatively by visual inspection of the representative treatments, and formal morphometric quantification (e.g., automated measures of cell shape such as circularity or confluence) was not performed. Accordingly, these morphology data are presented as supportive, descriptive evidence consistent with the quantified high-content imaging endpoints rather than as a standalone quantitative outcome.

## 4 Discussion

CKD represents one of the most common renal disorders in dogs, with progressive renal fibrosis serving as the final common pathway leading to end-stage renal disease. The TGF-β signaling pathway has been established as a central driver of renal fibrosis, making it an attractive therapeutic target for slowing the progression of CKD. Our findings support a model in which cirevetmab functionally targets a TGF-β1-dominant pro-fibrotic pathway in renal proximal tubular epithelial cells, with substantially reduced potency against TGF-β3 and no detectable functional neutralization of TGF-β2 under the conditions tested.

Renal fibrosis involves continuous repair marked by excessive accumulation of extracellular matrix (ECM) components. While normal ECM remodeling is essential for tissue health, uncontrolled and sustained ECM buildup becomes detrimental, eventually shifting from a protective to a destructive process, thus leading to chronic fibrosis. Another hallmark of the progression of fibrosis is epithelial to mesenchymal transition (EMT). During EMT, injured epithelial cells lose their tight junction proteins and acquire a mesenchymal phenotype. In the kidney, these processes contribute to the progressive decrease in glomerular filtration rate (GFR) and impairment of tubular function (28, 29). TGF-β signaling plays a critical role in this pathological process through multiple mechanisms such as activation of resident fibroblasts and myofibroblasts, induction of EMT in renal tubular epithelial cells, and direct stimulation of ECM deposition. The canonical TGF-β pathway represents the primary signaling mechanism through which TGF-β exerts these pro-fibrotic effects (7).

Consistent with canonical pathway activation, our data demonstrate that stimulation of renal proximal tubular epithelial cells with each of the three TGF-β isoforms induces SMAD2/3 phosphorylation and nuclear translocation. These data confirm that each ligand can engage the SMAD2/3 axis in this cell type and support its relevance as a target for therapeutic intervention.

However, despite sequence homology, the TGF-β isoforms are not functionally equivalent, and both expression patterns and downstream biological outcomes can differ. While TGF-β1 is the predominant isoform found within the kidney and is well studied for its role in renal fibrosis, TGF-β3 is less understood. It has been reported to exhibit context-dependent profibrotic and antifibrotic activities, complicating the interpretation of isoform-specific perturbation (30). Within this context, our findings highlight the isoform-selective modulation of SMAD2/3 signaling and fibrotic phenotypes by cirevetmab. Specifically, cirevetmab robustly blocks TGF-β1-induced SMAD2 nuclear translocation and αSMA/phalloidin fiber formation. In contrast, cirevetmab had minimal impact on TGF-β3 responses at the concentrations tested and no detectable effect on TGF-β2 -induced canonical signaling.

One data-consistent explanation for our observation that TGF-β3-driven fibrotic phenotypes are not altered with cirevetmab with partial neutralization of the SMAD signaling pathway is reduced functional potency against TGF-β3 rather than altered epitope binding. Given the high sequence homology among TGF-β isoforms and the monoclonal nature of cirevetmab, a major shift in binding epitope across ligands would not be expected. Instead, additional experiments (data not shown) indicate that increasing cirevetmab concentrations up to ~2 μM still do not inhibit TGF-β2-induced pSMAD signaling. In contrast, near complete neutralization of TGF-β3 pSMAD signaling was achievable at higher concentrations of cirevetmab, but with an IC_50_ value approximately 70-fold higher than observed for TGF-β1. Thus, isoform selectivity in this system appears to reflect a potency hierarchy rather than a binding phenomenon. An investigation into structural differences in epitope binding would be insightful to confirm this explanation but out of scope of this paper. It is reasonable to speculate that due to the known reduced potency of cirevetmab against TGF-β3-induced changes, increasing the concentration of antibody in these systems would show an effect on fibrotic markers. However, these experiments have not been performed, and other mechanistic conclusions cannot be ruled out.

Taken together, these findings suggest that the modest attenuation of SMAD2/3 signaling observed for TGF-β3 at the tested antibody concentration may be insufficient to shift downstream phenotypic endpoints, such as α-SMA, in this model. However, because we did not directly assess receptor complex dynamics or non-canonical pathway activity, additional mechanisms may also contribute to the discordance between SMAD pathway modulation and the lack of detectable phenotypic fibrotic changes.

In addition, several non-mutually exclusive mechanisms may contribute to the reduced apparent neutralization of TGF-β3 and/or to phenotype-level divergence from TGF-β1 in our model systems.

First, mechanistically, these potency differences may reflect ligand-specific structural and signaling properties that influence antibody access and/or receptor assembly. TGF-β3 has been described as adopting a more flexible “open” conformation relative to the more ordered structure of TGF-β1(31). Such differences could increase the antibody concentration required to effectively block functional receptor complex formation.

In addition, even when canonical SMAD signaling is activated, TGF-β1 versus TGF-β3-bound receptor complexes may differentially engage downstream signaling networks, raising the possibility that non-canonical pathways contribute to phenotype-level outputs in a manner not fully captured in our SMAD-driven readouts alone. Accordingly, investigation of the non-canonical signaling contributions in the context of cirevetmab activity is warranted to aid in fully interpreting pheontype-level responses.

The observed phenotype modulation is also relevant to canine CKD pathobiology. Because TGF-β is reported to influence multiple cell types involved in renal pathology, including mesangial cells in the glomerulus, fibroblasts, epithelial cells, and myofibroblasts and is widely implicated as a major driver of EMT in kidney disease, isoform-selective pathway modulation has potential implications for renal disease (13). In canine CKD, EMT is reflected by the loss of adhesion markers and increased expression of mesenchymal markers, both of which have been directly correlated with severity of tubulointerstitial disease (TID) (32). Tubulointerstitial nephritis is a common cause of CKD in canine patients with progression ultimately culminating in renal fibrosis (33, 34). Functionally, our findings show that cirevetmab preserves epithelial morphology and reduces α-SMA- positive fiber formation in proximal tubular epithelial cells following TGF-β1 stimulation. This suggests that targeting TGF-β1 may help mitigate EMT-related changes and early fibrotic remodeling that contribute to tubulointerstitial fibrosis. Notably, the degree of attenuation of TGF-β-driven phenotypes in canine cells was less pronounced than in human cells. This likely reflects experimental differences but may also indicate species-specific signaling thresholds or cellular context dependent pathway modulation. Regardless, the overall pattern in these data support the hypothesis that inhibition of TGF-β-mediated pathways, particularly TGF-β1-driven canonical signaling and fibrotic phenotypes, could reduce tubulointerstitial fibrosis in canine CKD, thereby potentially slowing disease progression.

Interpretation of these results should be done with several considerations. First, pSMAD2 translocation and αSMA/phalloidin fiber formation are robust but surrogate readouts for fibrosis; future work should quantify downstream transcriptional changes and extracellular matrix deposition to strengthen inference regarding antifibrotic effects. Second, dose-response relationships as they relate to ligand levels *in vivo* warrant deeper exploration to understand isoform selectivity and optimize exposure for canine systems. Third, additional studies in more physiologically relevant models (e.g., co-culture, 3D organoids, etc.) will be valuable to assess whether epithelial preservation persists with microenvironmental influences.

The study described demonstrates that a novel canine anti-TGF-β monoclonal antibody, cirevetmab, selectively neutralizes TGF-β1-, and to a lesser extent, TGF-β3-induced canonical SMAD signaling and nuclear translocation *in vitro*. Importantly cirevetmab specifically abrogates TGF-β1-induced fibrotic markers in these experimental systems. These findings collectively provide mechanistic support for targeting TGF-β1-dominant signaling and has potential to inform future *in vivo* studies as a strategy to mitigate renal fibrosis in canine CKD. While direct translation to canine disease and clinical efficacy cannot be established through these studies, our results provide valuable mechanistic insights into cirevetmab's action and lay the groundwork for further investigation to clarify TGF-β3 biology, non-canonical signaling and *in vivo* translatability to mitigate fibrotic disease progression in dogs.

## Data Availability

The original contributions presented in the study are included in the article/supplementary material, further inquiries can be directed to the corresponding author.

## References

[1] Bird LW. Pathophysiology of chronic kidney disease. Companion Anim. (2015) 20:15–9. doi: 10.12968/coan.2015.20.1.15

[2] Bartges JW. Chronic kidney disease in dogs and cats. Vet Clin North Am Small Anim Pract. (2012) 42:669-92, vi. doi: 10.1016/j.cvsm.2012.04.008 22720808

[3] O'Neill DG Elliott J Church DB McGreevy PD Thomson PC Brodbelt DC. Chronic kidney disease in dogs in UK veterinary practices: prevalence, risk factors, and survival. J Vet Intern Med. (2013) 27:814–21. doi: 10.1111/jvim.12090 23647231

[4] Thade GB Bhojne GR Dhoot VM. Successful Management of Chronic Kidney Disease in Dogs. Ind J Canine Pract. (2021) 13:54–7. doi: 10.29005/IJCP.2021.13.2.54-57

[5] Tarrant G Boyden L Rai T Wright A Cook AJC Wells K. Gaining insights into pet owner understanding/lived experience of canine chronic kidney disease using survey and social media data. Front Vet Sci. (2025) 12:1506272. doi: 10.3389/fvets.2025.1506272 40400671 PMC12094028

[6] Gu YY Liu XS Huang XR Yu XQ Lan HY. Diverse Role of TGF-beta in Kidney Disease. Front Cell Dev Biol. (2020) 8:123. doi: 10.3389/fcell.2020.00123 32258028 PMC7093020

[7] Meng XM Nikolic-Paterson DJ Lan HY. TGF-beta: the master regulator of fibrosis. Nat Rev Nephrol. (2016) 12:325–38. doi: 10.1038/nrneph.2016.48 27108839

[8] Ismail TA Farghali HA Khattab MS Ibrahem EM Sabry D ElMiniawy HMF. Development of Canine Chronic Kidney Disease Model: A Pilot Study. Int J Vet Sci. (2021) 10:286-93. doi: 10.47278/journal.ijvs/2021.056

[9] Huang R Fu P Ma L. Kidney fibrosis: from mechanisms to therapeutic medicines. Signal Transduct Target Ther. (2023) 8:129. doi: 10.1038/s41392-023-01379-7 36932062 PMC10023808

[10] Allinovi M De Chiara L Angelotti ML Becherucci F Romagnani P. Anti-fibrotic treatments: A review of clinical evidence. Matrix Biol. (2018) 68-69:333-54. doi: 10.1016/j.matbio.2018.02.017 29654884

[11] Mack M Yanagita M. Origin of myofibroblasts and cellular events triggering fibrosis. Kidney Int. (2015) 87:297–307. doi: 10.1038/ki.2014.287 25162398

[12] Nastase MV Zeng-Brouwers J Wygrecka M Schaefer L. Targeting renal fibrosis: Mechanisms and drug delivery systems. Adv Drug Deliv Rev. (2018) 129:295–307. doi: 10.1016/j.addr.2017.12.019 29288033

[13] Djudjaj S Boor P. Cellular and molecular mechanisms of kidney fibrosis. Mol Aspects Med. (2019) 65:16–36. doi: 10.1016/j.mam.2018.06.002 29909119

[14] Chen L Yang T Lu DW Zhao H Feng YL Chen H . Central role of dysregulation of TGF-beta/Smad in CKD progression and potential targets of its treatment. Biomed Pharmacother. (2018) 101:670–81. doi: 10.1016/j.biopha.2018.02.090 29518614

[15] Qi W Chen X Holian J Mreich E Twigg S Gilbert RE . Transforming growth factor-beta1 differentially mediates fibronectin and inflammatory cytokine expression in kidney tubular cells. Am J Physiol Renal Physiol. (2006) 291:F1070–7. doi: 10.1152/ajprenal.00013.2006 16720864

[16] Robertson IB Rifkin DB. Regulation of the Bioavailability of TGF-beta and TGF-beta-Related Proteins. Cold Spring Harb Perspect Biol. (2016) 8:a021907. doi: 10.1101/cshperspect.a021907 27252363 PMC4888822

[17] Tie Y Tang F Peng D Zhang Y Shi H. TGF-beta signal transduction: biology, function and therapy for diseases. Mol Biomed. (2022) 3:45. doi: 10.1186/s43556-022-00109-9 36534225 PMC9761655

[18] Chia ZJ Cao YN Little PJ Kamato D. Transforming growth factor-beta receptors: versatile mechanisms of ligand activation. Acta Pharmacol Sin. (2024) 45:1337–48. doi: 10.1038/s41401-024-01235-6 38351317 PMC11192764

[19] Deng Z Fan T Xiao C Tian H Zheng Y Li C . TGF-beta signaling in health, disease, and therapeutics. Signal Transduct Target Ther. (2024) 9:61. doi: 10.1038/s41392-024-01764-w 38514615 PMC10958066

[20] Sureshbabu A Muhsin SA Choi ME. TGF-beta signaling in the kidney: profibrotic and protective effects. Am J Physiol Renal Physiol. (2016) 310:F596–606. doi: 10.1152/ajprenal.00365.2015 26739888 PMC4824143

[21] Budi EH Duan D Derynck R. Transforming Growth Factor-beta Receptors and Smads: Regulatory Complexity and Functional Versatility. Trends Cell Biol. (2017) 27:658–72. doi: 10.1016/j.tcb.2017.04.005 28552280

[22] Li MO Wan YY Sanjabi S Robertson AK Flavell RA. Transforming growth factor-beta regulation of immune responses. Annu Rev Immunol. (2006) 24:99–146. doi: 10.1146/annurev.immunol.24.021605.090737 16551245

[23] Yu XY Sun Q Zhang YM Zou L Zhao YY. TGF-beta/Smad Signaling Pathway in Tubulointerstitial Fibrosis. Front Pharmacol. (2022) 13:860588. doi: 10.3389/fphar.2022.860588 35401211 PMC8987592

[24] Blobe GC Schiemann WP Lodish HF. Role of transforming growth factor beta in human disease. N Engl J Med. (2000) 342:1350–8. doi: 10.1056/NEJM200005043421807 10793168

[25] Tzavlaki K Moustakas A. TGF-β signaling. Biomolecules. (2020) 10:487. doi: 10.3390/biom10030487 32210029 PMC7175140

[26] Chen F Lyu L Xing C Chen Y Hu S Wang M . The pivotal role of TGF-beta/Smad pathway in fibrosis pathogenesis and treatment. Front Oncol. (2025) 15:1649179. doi: 10.3389/fonc.2025.1649179 40969268 PMC12440922

[27] Martinez-Hackert E Sundan A Holien T. Receptor binding competition: A paradigm for regulating TGF-beta family action. Cytokine Growth Factor Rev. (2021) 57:39–54. doi: 10.1016/j.cytogfr.2020.09.003 33087301 PMC7897244

[28] Weigle S Martin E Voegtle A Wahl B Schuler M. Primary cell-based phenotypic assays to pharmacologically and genetically study fibrotic diseases *in vitro*. J Biol Methods. (2019) 6:e115. doi: 10.14440/jbm.2019.285 31453262 PMC6706098

[29] Humphreys BD. Mechanisms of Renal Fibrosis. Annu Rev Physiol. (2018) 80:309–26. doi: 10.1146/annurev-physiol-022516-034227 29068765

[30] Escasany E Lanzón B García-Carrasco A Izquierdo-Lahuerta A Torres L Corrales P . Transforming growth factor beta3 deficiency promotes defective lipid metabolism and fibrosis in murine kidney. Dis Model Mech. (2021) 14:dmm.048249. doi: 10.1242/dmm.048249 34431499 PMC8489029

[31] Laverty HG Wakefield LM Occleston NL O'Kane S Ferguson MW. TGF-beta3 and cancer: a review. Cytokine Growth Factor Rev. (2009) 20:305–17. doi: 10.1016/j.cytogfr.2009.07.002 19656717 PMC7294566

[32] Benali SL Lees GE Castagnaro M Aresu L. Epithelial mesenchymal transition in the progression of renal disease in dogs. Histol Histopathol. (2014) 29:1409–14. 24872206 10.14670/HH-29.1409

[33] Im CY Kim SH Song KH Ryu MO Youn HY Seo KW. Pirfenidone inhibits TGF-beta1-induced fibrosis via downregulation of Smad and ERK pathway in MDCK cells. Vet Res Commun. (2024) 48:3167–76. doi: 10.1007/s11259-024-10493-y 39133399 PMC11442594

[34] Minkus G Reusch C Hörauf A Breuer W Darbès J Kraft W . Evaluation of renal biopsies in cats and dogs — histopathology in comparison with clinical data. J Small Anim Pract. (2008) 35:465–72. doi: 10.1111/j.1748-5827.1994.tb03952.x

